# Programme stoprisk: all about standard precautions

**DOI:** 10.1186/1753-6561-5-S6-P283

**Published:** 2011-06-29

**Authors:** S Monier, E Laprugne Garcia, M Giard, I Russell, J Fabry, A Savey

**Affiliations:** 1 CCLIN SUD EST, Saint Genis Laval, France

## Introduction / objectives

CCLIN Sud-Est offers a programme of innovative actions supporting the implementation of standard precautions: StopRisk is a quality and security improvement initiative for healthcare establishments. The programme started in 2009 and will continue until 2012. It answers two priority themes defined by the national programme of nosocomial infection prevention 2009/2013:

- promotion of a shared culture of quality and safety raising awareness and training of professionals.

- detection and pre-emption of emerging pathogenic agents by reinforcing prevention of cross transmission, in particular observance of standard precautions.

## Methods

To encourage training of personnel, communication aides and teaching tools are made available to healthcare establishments, training bodies and homes for the elderly. Three specific pages are thus freely accessible on the web. All aides are presented there, are regularly updated and may be downloaded.

The evaluation tool : self-evaluation audit was chosen to become a national audit in 2011.

Registered establishments benefit from the mailing of posters, leaflets and pamphlets. Completing a follow-up questionnaire allows these establishments to validate their participation.

## Results

User satisfaction was as much as 4.2 /5 for communication aides and 4.1/5 for teaching tools. Almost 8,000 professionals and more than 1,000 users took part, with an impact factor for the professionals of up to 3.7/5. 218 establishments have until now followed the programme, of which 98 have a validated participation.

## Conclusion

Standard precautions constitute the bedrock of risk reduction policy for personnel and patients. Training and awareness are considered as must-haves to support their observance. The StopRisk programme definitely helps healthcare establishments go this way.

## Disclosure of interest

None declared.

